# Elimination of Schistosomiasis Transmission in Zanzibar: Baseline Findings before the Onset of a Randomized Intervention Trial

**DOI:** 10.1371/journal.pntd.0002474

**Published:** 2013-10-17

**Authors:** Stefanie Knopp, Bobbie Person, Shaali M. Ame, Khalfan A. Mohammed, Said M. Ali, I. Simba Khamis, Muriel Rabone, Fiona Allan, Anouk Gouvras, Lynsey Blair, Alan Fenwick, Jürg Utzinger, David Rollinson

**Affiliations:** 1 Wolfson Wellcome Biomedical Laboratories, Department of Life Sciences, Natural History Museum, London, United Kingdom; 2 Department of Epidemiology and Public Health, Swiss Tropical and Public Health Institute, Basel, Switzerland; 3 University of Basel, Basel, Switzerland; 4 Schistosomiasis Consortium for Operational Research and Evaluation, Athens, Georgia, United States of America; 5 Public Health Laboratory - Ivo de Carneri, Pemba, United Republic of Tanzania; 6 Department of Infectious and Tropical Diseases, London School of Hygiene and Tropical Medicine, London, United Kingdom; 7 Helminth Control Laboratory Unguja, Ministry of Health, Zanzibar, United Republic of Tanzania; 8 Schistosomiasis Control Initiative, Department of Infectious Disease Epidemiology, Faculty of Medicine, London, United Kingdom; Centers for Disease Control and Prevention, United States of America

## Abstract

**Background:**

Gaining and sustaining control of schistosomiasis and, whenever feasible, achieving local elimination are the year 2020 targets set by the World Health Organization. In Zanzibar, various institutions and stakeholders have joined forces to eliminate urogenital schistosomiasis within 5 years. We report baseline findings before the onset of a randomized intervention trial designed to assess the differential impact of community-based praziquantel administration, snail control, and behavior change interventions.

**Methodology:**

In early 2012, a baseline parasitological survey was conducted in ∼20,000 people from 90 communities in Unguja and Pemba. Risk factors for schistosomiasis were assessed by administering a questionnaire to adults. In selected communities, local knowledge about schistosomiasis transmission and prevention was determined in focus group discussions and in-depths interviews. Intermediate host snails were collected and examined for shedding of cercariae.

**Principal Findings:**

The baseline *Schistosoma haematobium* prevalence in school children and adults was 4.3% (range: 0–19.7%) and 2.7% (range: 0–26.5%) in Unguja, and 8.9% (range: 0–31.8%) and 5.5% (range: 0–23.4%) in Pemba, respectively. Heavy infections were detected in 15.1% and 35.6% of the positive school children in Unguja and Pemba, respectively. Males were at higher risk than females (odds ratio (OR): 1.45; 95% confidence interval (CI): 1.03–2.03). Decreasing adult age (OR: 1.04; CI: 1.02–1.06), being born in Pemba (OR: 1.48; CI: 1.02–2.13) or Tanzania (OR: 2.36; CI: 1.16–4.78), and use of freshwater (OR: 2.15; CI: 1.53–3.03) showed higher odds of infection. Community knowledge about schistosomiasis was low. Only few infected *Bulinus* snails were found.

**Conclusions/Significance:**

The relatively low *S. haematobium* prevalence in Zanzibar is a promising starting point for elimination. However, there is a need to improve community knowledge about disease transmission and prevention. Control measures tailored to the local context, placing particular attention to hot-spot areas, high-risk groups, and individuals, will be necessary if elimination is to be achieved.

## Author Summary

Schistosomiasis is a chronic and debilitating disease caused by parasitic worms. It negatively impacts on the health and wellbeing of mainly rural dwellers in tropical and sub-tropical countries. The World Health Organization recently put forward an ambitious goal for the year 2020: to control schistosomiasis globally. Interruption of transmission and elimination of schistosomiasis are encouraged whenever resources allow. After careful consideration, the Schistosomiasis Consortium for Operational Research and Evaluation (SCORE) selected the Zanzibar archipelago to learn how best to eliminate schistosomiasis. We report the baseline findings of a 5-year program. Parasitological examination of about 20,000 people on Unguja and Pemba islands revealed a low overall prevalence of *Schistosoma haematobium* (7%). Nevertheless, hot-spots with high prevalence (>20%) and high-risk groups (males, young adults, people born in Pemba or mainland Tanzania, and people using natural freshwater) were identified. The community knowledge about schistosomiasis transmission and prevention was poor. Few of the collected intermediate host snails shed *S. haematobium* cercariae. A multi-arm randomized trial is now being implemented to determine the differential impact of mass deworming, snail control, and behavior change interventions. Lessons learned from this schistosomiasis elimination program will be important for other settings.

## Introduction

Schistosomiasis ranks third after soil-transmitted helminthiasis and leishmaniasis regarding disease burden estimates of neglected tropical diseases (NTDs), and causes an estimated 3.3 million disability-adjusted life years (DALYs) [1]. In Africa alone, it is estimated that some 200 million people are infected with the blood fluke of the genus *Schistosoma* [2].

Encouragingly, over the past decade, efforts to control NTDs have been scaled up [3]. In early 2012, the World Health Organization (WHO) issued an ambitious goal to control schistosomiasis globally by the year 2020 and put forward a roadmap as to how this could be achieved [4]. A number of influential public and private organizations now support this goal and contributed to the London Declaration [5]. In May 2012, the World Health Assembly (WHA) resolution 65.21 was adopted, which encourages member states and the international community not only to make available the necessary and sufficient means and resources in terms of medicines, but also in terms of water, sanitation, and hygiene interventions [6].

While preventive chemotherapy is considered as the mainstay of schistosomiasis control [7]–[9], there is considerable evidence that control packages integrating anti-schistosomal treatment, the provision of clean water and improved sanitation, snail control, and behavior change, readily adapted to the local settings and fine-tuned over time, are necessary to sustain control achievements and to reach elimination of schistosomiasis [10]–[13]. Political will and support from national governments, institutions, and the local population coupled with inter-sectoral collaboration between the health, water and sanitation, and education sectors are key features to achieve sustainable control of schistosomiasis [14]–[17]. Examples of where schistosomiasis has been successfully controlled or even eliminated using integrated measures include, besides others, Japan and the People's Republic of China (*S. japonicum*), Martinique and Saudi Arabia (*S. mansoni*), and Tunisia and Mauritius (*S. haematobium*) [12]. The Zanzibar archipelago, part of the United Republic of Tanzania, has been identified as a candidate area, where schistosomiasis elimination might be achieved [4], [18]–[20]. Indeed, after careful consideration, the Schistosomiasis Consortium for Operational Research and Evaluation (SCORE), selected the Zanzibar archipelago to learn how best to eliminate schistosomiasis and to evaluate different intervention combinations. Selection criteria included (i) the strong political commitment from the Zanzibar President and the government; (ii) the restriction to only urogenital schistosomiasis caused by *S. haematobium*; (iii) the relatively low *S. haematobium* prevalence and infection intensity on both islands; and (iv) the creation of an alliance determined to achieve schistosomiasis elimination in Zanzibar. This alliance – Zanzibar Elimination of Schistosomiasis Transmission (ZEST) – was formed in 2011 and consists of the Zanzibar government, particularly the Ministries of Health and Education, the Public Health Laboratory – Ivo de Carneri (PHL–IdC) Pemba, and a growing number of partners, including SCORE, Natural History Museum (NHM) in London, WHO, Schistosomiasis Control Initiative (SCI), Swiss Tropical and Public Health Institute (Swiss TPH), and other institutions and individuals. ZEST aims at (i) eliminating schistosomiasis as a public health problem on Unguja island in 3 years and to interrupt transmission in 5 years; (ii) controlling schistosomiasis throughout Pemba island (prevalence <10%) in 3 years and eliminating it as a public health problem in 5 years; and (iii) gaining experiences and drawing lessons for successful and durable schistosomiasis control, including costs and barriers associated with three different control interventions. Elimination of schistosomiasis as a public health problem is defined as the reduction of the prevalence of *S. haematobium* to <1% heavy infections based upon direct egg-detection methods in the school-aged population [12].

Here, we describe the baseline characteristics of local communities in Zanzibar, prior to the implementation of a randomized intervention trial, that consists of biannual mass drug administration (MDA) of praziquantel to the whole at-risk population (arm 1), compared to MDA plus snail control interventions (arm 2), and to MDA plus behavior change interventions (arm 3) [19]. Challenges and opportunities are discussed.

## Methods

### Ethics Statement

The study protocol received ethical approval from the Zanzibar Medical Research Ethics Committee (ZAMREC, reference no. ZAMREC 0003/Sept/011), the “Ethikkomission beider Basel” (EKBB) in Switzerland (reference no. 236/11), and the Institutional Review Board of the University of Georgia (project no. 2012-10138-0). Formative research on behavior change interventions was approved by the National Center for Emerging Zoonotic Diseases (NCEZID) of the Centers for Disease Control and Prevention (NCEZID tracking no. 103111BP). The study is designed as randomized intervention trial and is registered at the International Standard Randomised Controlled Trial Number Register (ISRCTN48837681).

The purpose and procedures of the study were verbally explained to village and school authorities and to study participants. Participants received an information sheet and were asked to submit a written informed consent. All minors (e.g., children below the age of 16 years) included into the study had written informed consent given by their parents/guardians and all participating adults signed and provided their own consent.

All participants were offered praziquantel (40 mg/kg) against schistosomiasis and albendazole (400 mg) against soil-transmitted helminthiasis free of charge in the frame of the island-wide MDA campaign conducted in late April 2012.

### Study Area and Population

The Zanzibar archipelago includes the two large islands of Unguja and Pemba. Unguja is divided into six and Pemba into four districts, which are further subdivided into smaller administrative units, known as shehias. The local administration in the shehias is governed by the community leader (sheha). According to the 2002 census, Unguja consists of 176 and Pemba of 73 shehias with a total population of 979,637 inhabitants. The mean annual growth rate is 3.1%, and hence, the estimated population in 2012 was 1,330,000. The majority of the population is Muslim.

For inclusion into our intervention trial, we randomly selected 45 shehias in both Unguja and Pemba [19]. The three intervention arms (i.e., MDA alone, MDA plus snail control, and MDA plus behavior change) will be monitored longitudinally in 15 shehias each, on both islands, by means of annual cross-sectional parasitological surveys together with snail surveys, and qualitative behavioral assessments [19].

### Field Procedures

Details of the study surveys are provided elsewhere [19]. In brief, before the onset of regular biannual MDA in April 2012, we conducted a baseline parasitological survey assessing *S. haematobium* infection in adults and school children. The adult survey was conducted in November and December 2011. In each study shehia, the sheha was invited to answer a set of standard questions about the demographics, sanitary infrastructure, and water availability and use in the shehia. Moreover, 50 randomly selected households in each shehia were visited by a member of a 4–12 headed trained interviewer team. In each household, one present adult, aged 20–55 years, was randomly selected, informed about the ZEST program, and asked for consent to participate. Participation included answering standard questions about demographics such as age, occupation, and risk factors potentially associated with *S. haematobium* transmission. Moreover, all participants were invited to submit a urine sample right after the questionnaire interview (between 09:00 and 18:00 hours).

The baseline survey in 45 primary schools was conducted from January to March 2012. A school was visited on two subsequent days by two field teams consisting of 2–4 fieldworkers for registration and 4–6 fieldworkers for sample collection, respectively. On the first visit, approximately 130 children attending standard 1 and 130 children from standards 3 and 4 were stratified by sex and randomly selected for participation. Children's names and demographic details were registered. After explaining the study purpose and procedures, children were provided with a consent sheet to be signed by the parents/guardians and to be returned the next day. Upon submission of the signed informed consent sheet, a urine sample was collected from children attending standards 1, 3, and 4 (between 09:00 and 14:00 hours).

Urine samples from adults and school children were transferred to the laboratory in Zanzibar Town (Helminth Control Laboratory Unguja, HCLU) or Chake (PHL–IdC) immediately after collection.

### Laboratory Procedures

Urine samples were processed either the same day (HCLU) or stored in the fridge until the next morning (PHL–IdC). Urine samples of sufficient quantity (≥10 ml) were visually inspected for blood (macrohematuria) using a color chart, for microhematuria using reagent strips (Hemastix; Siemens Healthcare Diagnostics GmbH, Eschborn, Germany), and for *S. haematobium* eggs by filtering 10 ml of urine through a polycarbonate filter (Sterlitech, Kent, United States of America) that was quantitatively examined under a microscope by experienced laboratory technicians.

### Snail Survey

In Zanzibar, four species of *Bulinus* spp. snails are recognized [21], [22]. While *B. globosus* and *B. nasutus* are allopatric, *B. forskalii* may be found in association with both species. *Bulinus* sp., another *B. forskalii* group species, has a very limited distribution [22]. In Unguja, *B. nasutus* only occurs in the south of the island in areas that are not part of our survey. We are hence only dealing with *B. globosus* and *B. forskalii* in our surveyed shehias in Unguja. In Pemba, however, the distribution lines of *B. globosus* and *B. nasutus* are not as clear cut. Since it is difficult to differentiate the taxa of *B. globosus* and *B. nasutus* using shell characteristics, we refer in the following to *B. globosus*/*nasutus* for snails collected in Unguja or Pemba. It must be noted, however, that in Zanzibar only *B. globosus* seems susceptible for *S. haematobium* miracidia infection [23]. The snail control arm in our study aims to minimize schistosomiasis transmission by reducing intermediate host snail populations of *B. globosus* by the use of the molluscicide niclosamide [19]. Before the onset of niclosamide application, between November 2011 and December 2012, freshwater bodies in the 15 randomly selected shehias on each island were identified with the help of local people and mapped using a hand-held global positioning system (Garmin GPSMap 62, Garmin Ltd., Southhampton, United Kingdom). Water characteristics such as conductivity, dissolved oxygen, pH, salinity, temperature, total dissolved solids (TDS), and velocity were measured and recorded with a hand-held water meter (PcTest 35K, Thermo Fisher Scientific Inc., Loughborough, United Kingdom). To assess snail densities, a sample area of 15 m was measured and subsequently surveyed for snails for 15 min by four trained staff using scoops, sieves, and hands. The collected snails per site were placed into one collecting tray, identified at genus level, and counted on the spot. Once the species and number of snails were recorded, all organisms, except *Bulinus* spp., were returned and evenly distributed to their original habitats. All *B. globosus*/*nasutus* were transferred to HCLU or PHL–IdC to observe shedding of cercariae and all *B. forskalii* for more detailed species investigations.

In the laboratory, *B. globosus*/*nasutus* snails were assessed the following day for *S. haematobium* infection by placing snails individually into a flat-bottom vial, in clean water, exposing the snails to sun light for 2 hours and subsequently observing cercariae shed using a dissection microscope [24], [25]. *S. haematobium* cercariae were identified microscopically by experienced technicians of the “snail team” and captured on Whatman FTA classic cards (Whatman, Part of GE Healthcare, Florham Park, United States of America) to identify molecular characteristics at a later point in time [26].

### Formative Research for Behavior Change

The behavior change study arm aims to interrupt the transmission cycle of *S. haematobium* by modifying people's behavior [19]. To identify and implement community-owned behavior change interventions, we follow a ‘Human-centered Design Process’ [27]. This process creates solutions to problems in joint collaboration with the community. The first step was to conduct qualitative formative research with study community members, in five shehias on Unguja (Chaani, Dole, Kilombero, Mwera, and Uzini) and two shehias on Pemba (Chambani and Kizimbani), among the 15 selected study shehias on each island. We explored community perceptions and practices associated with transmitting, having, treating and preventing schistosomiasis, locally known as *kichocho*. Community leaders, religious leaders, teachers, parents and school children were asked for their opinion in focus group discussions (FGDs) and in-depth interviews (IDIs) conducted in Kiswahili by trained members of the “behavioral team” of the HCLU and PHL–IdC in 2011.

### Statistical Analysis

Registration details and quantitative data from laboratory examinations were entered in Microsoft Excel version 10.0 (2002 Microsoft Corporation) and snail records and questionnaire data into EpiInfo version 3.5.1 (Centers for Disease Control and Prevention, Atlanta, United States of America) by local staff in Zanzibar. Statistical analyses were carried out with STATA version 10 (StataCorp., College Station, United States of America). FGDs and IDIs were transcribed verbatim, translated from Kiswahili into English, and entered into Atlas-ti version 6.0 (Software Development GmbH, Berlin, Germany) [19]. Sections of narrative data were open-coded to create specific categories and axial-coding was employed to relate the categories to each other. A thematic analysis was conducted through a grounded theory framework. Sets of codes were integrated and emergent themes identified.

Into the analyses of parasitological and questionnaire data only adults aged 20–55 years, first-year students and children aged 9–12 years were included, adhering to SCORE guidelines [19]. Macrohematuria was graded with numbers from 1 to 6 from transparent to dark red urine using a pretested color chart [28], [29]. Microhematuria in urine was coded semi-quantitatively according to the Hemastix manufacturer's instructions (0, negative; 1, +; 2, ++; 3, +++; and 4, trace). *S. haematobium* infection intensity was determined according to the number of eggs found in a 10 ml filtrate with 1–49 eggs/10 ml considered as light and ≥50 eggs/10 ml as heavy infections [30]. Association between *S. haematobium* infection (binary variable) or egg counts per 10 ml urine (continuous variable) and macro- or microhematuria (categorical variables) was assessed by univariable logistic regression.

Stratified by island, univariable and stepwise backward multivariable regression analyses were employed to identify significant associations between *S. haematobium* infection and risk factors, as assessed in the questionnaire interviews with adults. Candidate explanatory variables for the multivariable logistic regression were sex (binary variable) and age (continuous variable) and those which were significantly associated with *S. haematobium* infection in the univariable analyses. In the backward stepwise multivariable logistic regression, we removed non-predicting covariates up to a significance level of 0.2 and allowed for possible clustering by using the sandwich estimator robust cluster option in STATA. Similarly, the association between *Bulinus* spp. snails and water chemistry parameters (conductivity, pH, temperature, and TDS: continuous variables; velocity: ordinary variable) were analyzed using univariable and multivariable regression, including only covariables that were significant in univariable analysis.

## Results

### Study Compliance

**Figure 1:**
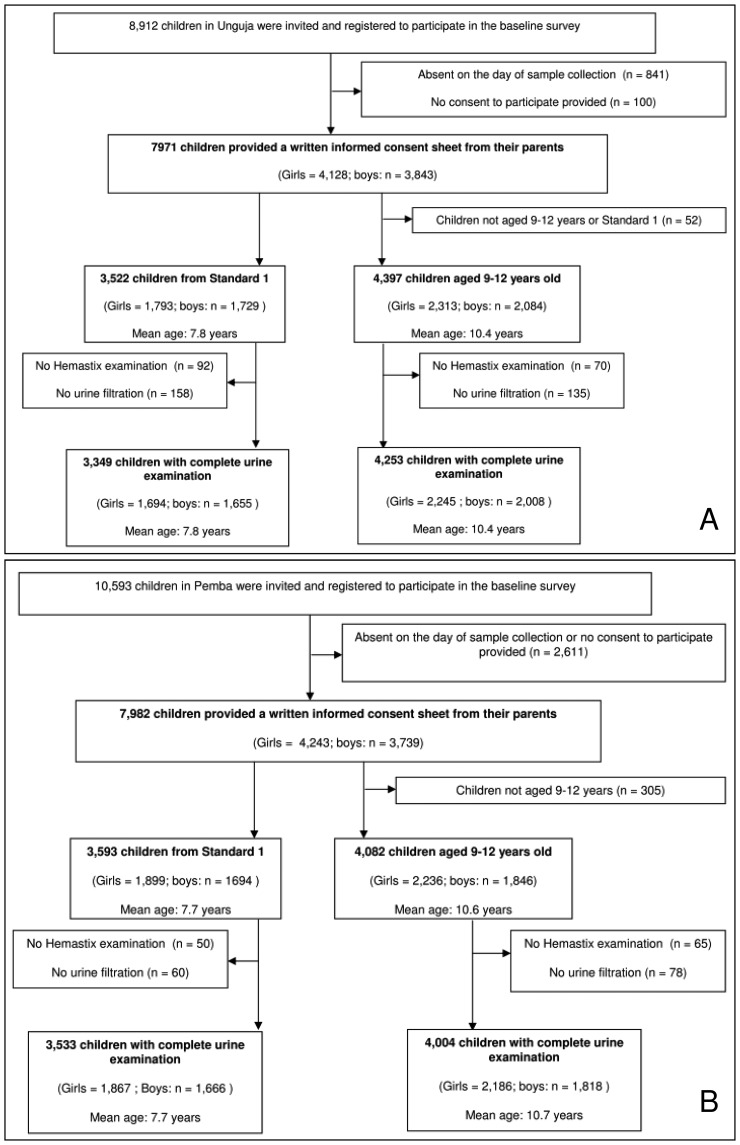
Flowchart detailing study participation in the schools surveyed in Unguja (A) and Pemba (B) in January till March, 2012.

The shehas of all 90 study shehias agreed to answer the questions about shehia characteristics. Both on Unguja and Pemba, 2,250 adult individuals were invited to participate in the study and provided written informed consent. In Unguja, 2,196 and in Pemba 1,867 adults were in the 20–55 years age range, and hence included in the analyses. On both islands, the adults' mean age was 34 years. In Unguja 1,670 (76.1%) and in Pemba 1,242 (66.6%) participants were female. All participants replied at least partially to the questionnaire survey. The number of individuals who provided a urine sample of sufficient quantity for hematuria assessment and urine filtration is shown in Table 1. The compliance of the 8,912 and 10,593 school children, who were invited to participate in the study in Unguja and Pemba, respectively, is detailed in Figure 1.

**Table 1:** *S. haematobium* infection characteristics stratified by survey group.

Study participants	Infection characteristics	Unguja			Pemba		
		n	n pos	%	n	n pos	%
**Children (1st year)**	Hemastix hematuria	3,430			3,543		
	0		3,313	96.6		3,014	85.1
	Trace		3	0.1		157	4.4
	+		23	0.7		78	2.2
	++		38	1.1		167	4.7
	+++		53	1.5		127	3.6
	*S. haematobium* eggs	3,364	174	5.2	3,533	432	12.2
	Low infection intensity		142	81.6		258	59.7
	High infection intensity		32	18.4		163	37.7
**Children (9–12 years)**	Hemastix hematuria	4,327			4,017		
	0		4,011	92.7		3,572	88.9
	Trace		11	0.3		134	3.3
	+		98	2.3		105	2.6
	++		94	2.2		110	2.7
	+++		113	2.6		96	2.4
	*S. haematobium* eggs	4,262	164	3.8	4,004	326	8.1
	Low infection intensity		145	88.4		219	67.2
	High infection intensity		19	11.6		107	32.8
**Adults (20–55 years)**	Hemastix hematuria	2,155			1,864		
	0		1,931	89.6		1,598	85.7
	Trace		50	2.3		84	4.5
	+		55	2.6		86	4.6
	++		52	2.4		67	3.6
	+++		67	3.1		29	1.6
	*S. haematobium* eggs	2,134	57	2.7	1,861	102	5.5
	Low infection intensity		53	93.0		95	93.1
	High infection intensity		4	7.0		7	6.9

### Shehia Characteristics

On average, a shehia on Unguja and Pemba has a size of 9.5 km^2^ and 13.5 km^2^, respectively. In Unguja, the population in the 45 study shehias ranged from 880 in the rural shehia Donge Mnyimbi to 15,000 in the urban shehia Melinne. In 2011, on average, 19 (range: 0–186) people immigrated and 10 (range: 0–50) emigrated per shehia. Rice farming (62.9%), vegetable farming (17.1%), and banana farming (11.4%) were the activities that most shehas attributed as primary occupation related to being exposed to open freshwater bodies in their shehia. Natural freshwater was used as optional drinking, washing, or bathing water in 24.4%, 28.9%, and 28.9% of shehias, respectively. A number of shehas also reported the implementation of new wells (11 shehias), new taps (eight shehias), new polytanks (five shehias), new rainwater tanks (three shehias), new household toilets (40 shehias), and new public toilets (17 shehias) in their shehia in 2011.

In Pemba, between 2,043 (Makombeni) and 12,781 (Msuka) people inhabited a shehia. On average, 16 (range: 0–60) immigrants and nine (range: 0–30) emigrants per shehia were counted by the shehas in 2011. Shehas mentioned rice farming as the predominant agricultural activity involving freshwater contact (88.9%). Natural freshwater was not mentioned as drinking water source in Pemba, but 77.8% and 75.6% of shehas reported the use of natural freshwater for bathing or washing, respectively. In some shehias, new wells (four shehias), new taps (one shehia), new household toilets (42 shehias), and new public toilets (six shehias) were implemented in 2011.

### *S. haematobium* Infection Characteristics, Stratified by Sentinel Group

**Figure 2:**
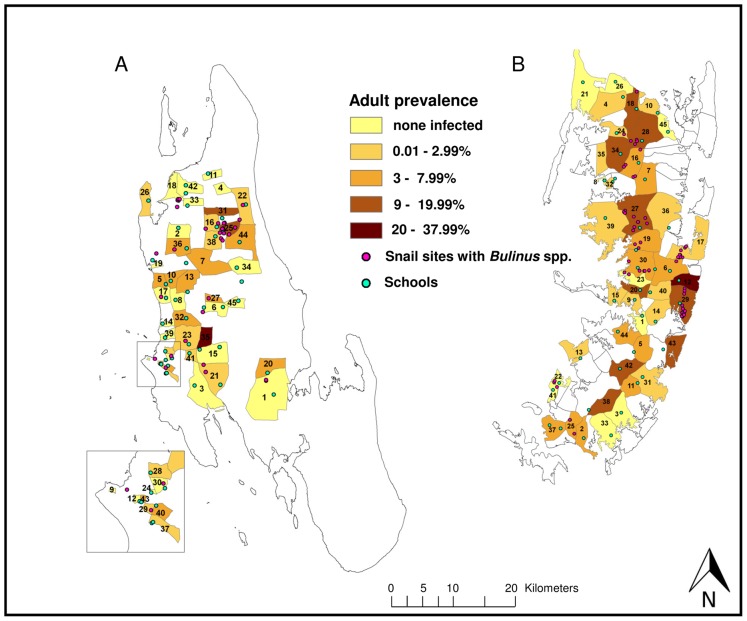
*S. haematobium* prevalence in adults in 45 study shehias in Unguja (A) and Pemba (B). Map indicating the *S. haematobium* prevalence in the adult population of the 45 study shehias in Unguja (A) and Pemba (B) at the baseline survey conducted in November/December 2011, surveyed schools in all 45 study shehias per island and sites in the 15 snail control shehias per island, where *Bulinus* spp. snails were found. Shehias in Unguja and their assigned intervention: Biannual mass drug administration with praziquantel: 1 = Cheju, 2 = Donge Mnyimbi, 3 = Fuoni Kibondeni, 4 = Gamba, 5 = Kama, 6 = Kiboje Mkwajuni, 7 = Kitope, 8 = Mbuzini, 9 = Mchangani, 10 = Mfenesini, 11 = Mkwajuni, 12 = Muungano, 13 = Mwakaje, 14 = Mwanyanya, 15 = Ubago; biannual mass drug administration with praziquantel plus snail control: 16 = Bandamaji, 17 = Chuini, 18 = Donge Mchangani, 19 = Fujoni, 20 = Jendele, 21 = Jumbi, 22 = Kandwi, 23 = Kianga, 24 = Kilimahewa Juu, 25 = Kinyasini, 26 = Mafufuni, 27 = Miwani, 28 = Mtopepo, 29 = Nyerere, 30 = Welezo; biannual mass drug administration with praziquantel plus behaviour change interventions: 31 = Chaani Kubwa, 32 = Dole, 33 = Donge Mtambile, 34 = Kilombero, 35 = Koani, 36 = Mahonda, 37 = Melinne, 38 = Mgambo, 39 = Mtoni, 40 = Mwanakwerekwe, 41 = Mwera, 42 = Pale, 43 = Sebleni, 44 = Upenja, 45 = Uzini. Shehias in Pemba and their assigned intervention: Biannual mass drug administration with praziquantel: 1 = Chanjaani, 2 = Kangani, 3 = Kiwani, 4 = Konde, 5 = Matale, 6 = Ole, 7 = Pandani, 8 = Selemu, 9 = Tibirinzi, 10 = Tumbe, 11 = Ukutini, 12 = Uwandani, 13 = Wambaa, 14 = Wawi, 15 = Wesha; Biannual mass drug administration with praziquantel plus snail control: 16 = Finya,17 = Kangagani, 18 = Kinowe, 19 = Kisiwani, 20 = Kwale, 21 = Makangale, 22 = Makombeni, 23 = Mbuzini, 24 = Mgogoni, 25 = Mkanyageni, 26 = Msuka, 27 = Piki, 28 = Shumba Viamboni, 29 = Vitongoji, 30 = Ziwani; biannual mass drug administration with praziquantel plus behaviour change interventions: 31 = Chambani, 32 = Jadida, 33 = Kengeja, 34 = Kinyasini, 35 = Kizimbani, 36 = Mchangamdogo, 37 = Michenzani, 38 = Mtambile, 39 = Mtambwe Kusini, 40 = Ng'ambwa, 41 = Ng'ombeni, 42 = Ngwachani, 43 = Pujini, 44 = Shungi, 45 = Sizini.

Microhematuria was detected in the urine of 10.4% and 14.3% of surveyed adults in Unguja and Pemba, respectively (Table 1). The respective *S. haematobium* infection prevalence in this age group was 2.7% and 5.5%. The highest *S. haematobium* prevalence in adults was found in the shehia Koani (26.5%) in Unguja, and in the shehia Uwandani (23.4%) in Pemba (Figure 2).

**Figure 3:**
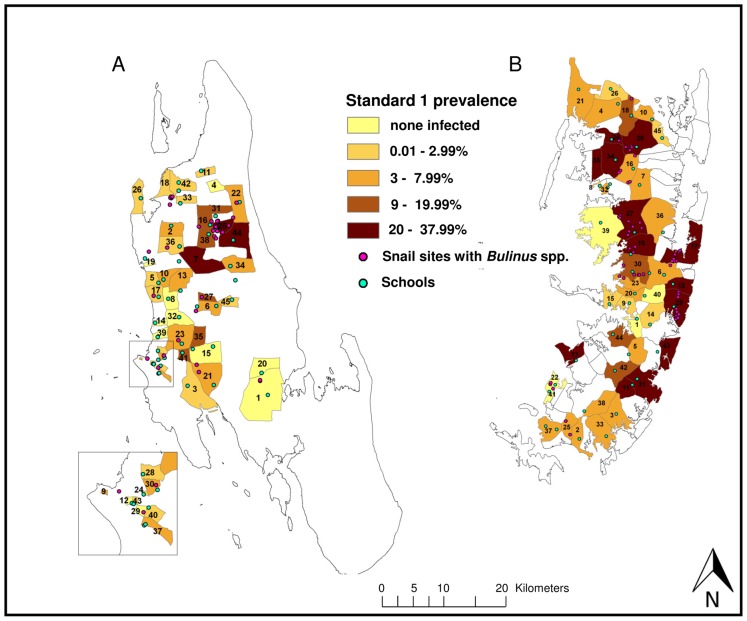
*S. haematobium* prevalence in first-year school children in 45 study shehias in Unguja (A) and Pemba (B). Map indicating the *S. haematobium* prevalence in first-year school children in the 45 study shehias in Unguja (A) and Pemba (B) at the baseline survey conducted in January-March 2012, surveyed schools in all 45 study shehias per island and sites in the 15 snail control shehias per island, where *Bulinus* spp. snails were found. Shehias in Unguja and their assigned intervention: Biannual mass drug administration with praziquantel: 1 = Cheju, 2 = Donge Mnyimbi, 3 = Fuoni Kibondeni, 4 = Gamba, 5 = Kama, 6 = Kiboje Mkwajuni, 7 = Kitope, 8 = Mbuzini, 9 = Mchangani, 10 = Mfenesini, 11 = Mkwajuni, 12 = Muungano, 13 = Mwakaje, 14 = Mwanyanya, 15 = Ubago; biannual mass drug administration with praziquantel plus snail control: 16 = Bandamaji, 17 = Chuini, 18 = Donge Mchangani, 19 = Fujoni, 20 = Jendele, 21 = Jumbi, 22 = Kandwi, 23 = Kianga, 24 = Kilimahewa Juu, 25 = Kinyasini, 26 = Mafufuni, 27 = Miwani, 28 = Mtopepo, 29 = Nyerere, 30 = Welezo; biannual mass drug administration with praziquantel plus behaviour change interventions: 31 = Chaani Kubwa, 32 = Dole, 33 = Donge Mtambile, 34 = Kilombero, 35 = Koani, 36 = Mahonda, 37 = Melinne, 38 = Mgambo, 39 = Mtoni, 40 = Mwanakwerekwe, 41 = Mwera, 42 = Pale, 43 = Sebleni, 44 = Upenja, 45 = Uzini. Shehias in Pemba and their assigned intervention: Biannual mass drug administration with praziquantel: 1 = Chanjaani, 2 = Kangani, 3 = Kiwani, 4 = Konde, 5 = Matale, 6 = Ole, 7 = Pandani, 8 = Selemu, 9 = Tibirinzi, 10 = Tumbe, 11 = Ukutini, 12 = Uwandani, 13 = Wambaa, 14 = Wawi, 15 = Wesha; Biannual mass drug administration with praziquantel plus snail control: 16 = Finya,17 = Kangagani, 18 = Kinowe, 19 = Kisiwani, 20 = Kwale, 21 = Makangale, 22 = Makombeni, 23 = Mbuzini, 24 = Mgogoni, 25 = Mkanyageni, 26 = Msuka, 27 = Piki, 28 = Shumba Viamboni, 29 = Vitongoji, 30 = Ziwani; biannual mass drug administration with praziquantel plus behaviour change interventions: 31 = Chambani, 32 = Jadida, 33 = Kengeja, 34 = Kinyasini, 35 = Kizimbani, 36 = Mchangamdogo, 37 = Michenzani, 38 = Mtambile, 39 = Mtambwe Kusini, 40 = Ng'ambwa, 41 = Ng'ombeni, 42 = Ngwachani, 43 = Pujini, 44 = Shungi, 45 = Sizini.

**Figure 4:**
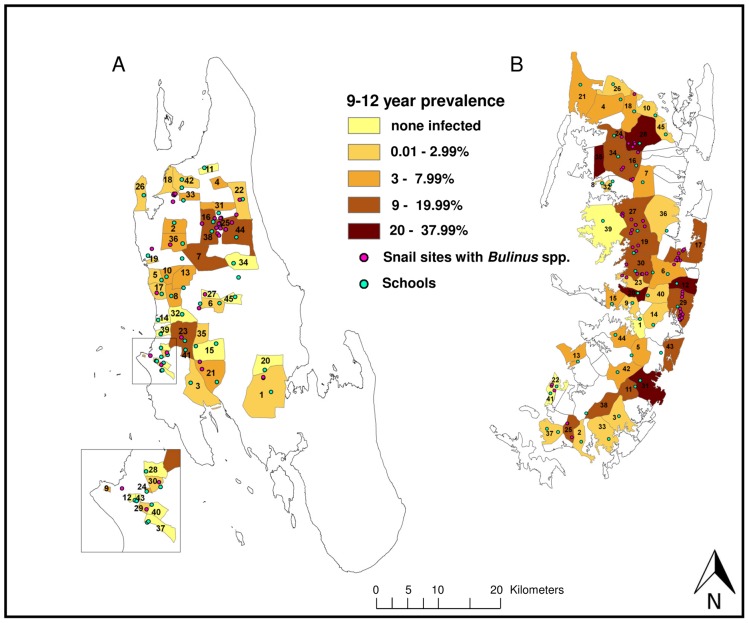
*S. haematobium* prevalence in 9–12-year-old school children in 45 study shehias in Unguja (A) and Pemba (B). Map indicating the *S. haematobium* prevalence in 9- to 12-year school children in the 45 study shehias in Unguja (A) and Pemba (B) at the baseline survey conducted in January-March 2012, surveyed schools in all 45 study shehias per island and sites in the 15 snail control shehias per island, where *Bulinus* spp. snails were found. Shehias in Unguja and their assigned intervention: Biannual mass drug administration with praziquantel: 1 = Cheju, 2 = Donge Mnyimbi, 3 = Fuoni Kibondeni, 4 = Gamba, 5 = Kama, 6 = Kiboje Mkwajuni, 7 = Kitope, 8 = Mbuzini, 9 = Mchangani, 10 = Mfenesini, 11 = Mkwajuni, 12 = Muungano, 13 = Mwakaje, 14 = Mwanyanya, 15 = Ubago; biannual mass drug administration with praziquantel plus snail control: 16 = Bandamaji, 17 = Chuini, 18 = Donge Mchangani, 19 = Fujoni, 20 = Jendele, 21 = Jumbi, 22 = Kandwi, 23 = Kianga, 24 = Kilimahewa Juu, 25 = Kinyasini, 26 = Mafufuni, 27 = Miwani, 28 = Mtopepo, 29 = Nyerere, 30 = Welezo; biannual mass drug administration with praziquantel plus behaviour change interventions: 31 = Chaani Kubwa, 32 = Dole, 33 = Donge Mtambile, 34 = Kilombero, 35 = Koani, 36 = Mahonda, 37 = Melinne, 38 = Mgambo, 39 = Mtoni, 40 = Mwanakwerekwe, 41 = Mwera, 42 = Pale, 43 = Sebleni, 44 = Upenja, 45 = Uzini. Shehias in Pemba and their assigned intervention: Biannual mass drug administration with praziquantel: 1 = Chanjaani, 2 = Kangani, 3 = Kiwani, 4 = Konde, 5 = Matale, 6 = Ole, 7 = Pandani, 8 = Selemu, 9 = Tibirinzi, 10 = Tumbe, 11 = Ukutini, 12 = Uwandani, 13 = Wambaa, 14 = Wawi, 15 = Wesha; Biannual mass drug administration with praziquantel plus snail control: 16 = Finya,17 = Kangagani, 18 = Kinowe, 19 = Kisiwani, 20 = Kwale, 21 = Makangale, 22 = Makombeni, 23 = Mbuzini, 24 = Mgogoni, 25 = Mkanyageni, 26 = Msuka, 27 = Piki, 28 = Shumba Viamboni, 29 = Vitongoji, 30 = Ziwani; biannual mass drug administration with praziquantel plus behaviour change interventions: 31 = Chambani, 32 = Jadida, 33 = Kengeja, 34 = Kinyasini, 35 = Kizimbani, 36 = Mchangamdogo, 37 = Michenzani, 38 = Mtambile, 39 = Mtambwe Kusini, 40 = Ng'ambwa, 41 = Ng'ombeni, 42 = Ngwachani, 43 = Pujini, 44 = Shungi, 45 = Sizini.

In Unguja, 3.4% of the children from standard 1 had microhematuria and 5.2% had *S. haematobium* eggs diagnosed in their urine. Microhematuria was detected in 7.3% and *S. haematobium* infections in 3.8% of the school children aged 9–12 years. The highest *S. haematobium* prevalence of 26.8% in standard 1 children was found in the shehia Upenja (Figure 3) and of 20.0% in 9- to 12-year-old children from the shehia Kinyasini (Figure 4).

In Pemba, microhematuria and *S. haematobium* eggs in urine were diagnosed in 14.9% and 12.2% of children attending standard 1 and in 11.1% and 8.1% of school children aged 9–12 years, respectively. The highest prevalence in standard 1 and among 9- to 12-year-old children was observed in the shehias Uwandani (37.0%) and Kizimbani (29.0%), respectively.

As detailed in Table 2, macro- and microhematuria were strongly associated with *S. haematobium* infection in adults and school children, both in Unguja and Pemba. Children and adults with eggs identified in their urine had significantly higher odds of a trace, +, ++, or +++ result indicated by the reagent strip. Combining all data from adults and children and both islands, we found a significant correlation between the number of eggs detected in 10 ml urine and the color grading for macrohematuria (odds ratio (OR): 1.24, 95% confidence interval (CI): 1.07–1.21) and microhematuria (OR: 3.32, 95% CI: 3.17–3.48).

**Table 2:** Association between *S. haematobium* infection and macro- and microhematuria.

Study participants	Infection characteristics	Variable	Unguja			Pemba		
			n	OR	95% CI	n	OR	95% CI
**Adults (20–55 years)**	Visible hematuria	continuous	2,133	1.8	1.3–2.5	1,860	1.3	1.01–1.6
	Hemastix hematuria	Trace	2,133	8.4	3.1–22.9	1,860	10.8	5.7–20.5
		+		2.8	0.7–12.3		10.5	5.5–19.9
		++		15.8	7.0–35.8		21.0	11.3–38.9
		+++		23.6	11.9–47.0		49.2	22.0–109.8
**Children (9–12 years)**	Visible hematuria	continuous	4,253	1.8	1.5–2.3	4,004	2.0	1.7–2.3
	Hemastix hematuria	Trace	4,242	NA	NA	4,004	126.9	77.4–208.1
		+		6.8	3.7–12.4		245.8	142.6–423.6
		++		7.6	4.2–13.6		223	130.9–379.8
		+++		18.8	12.1–29.4		898.8	435.8–1853.8
**Children (1st year)**	Visible haematuria	continuous	3349	1.1	0.111	3533	2.2	<0.001
	Hemastix haematuria	trace	3349	12.7	0.039	3533	438.9	<0.001
		1		5.6	<0.001		624.5	<0.001
		2		21.5	<0.001		1247.7	<0.001
		3		29.5	<0.001		3016.6	<0.001

CI: confidence interval.

OR = odds ratio.

NA = not applicable.

### Risk Factors for *S. haematobium* Infection

In Unguja, males (OR: 2.56, 95% CI: 1.50–4.35), people of young adult age (OR: 1.05; 95% CI: 1.02–1.08), Christians (OR: 3.67, 95% CI: 1.41–9.56), those born in mainland Tanzania (OR: 2.51; 95% CI: 1.24–5.08), and those using natural freshwater (OR: 2.09, 95% CI: 1.11–3.94) had an elevated risk of *S. haematobium* infection according to univariable regression analysis. With the exception of religion, these explanatories remained significant also in the adjusted multivariable regression analyses (n = 2,131).

In Pemba, young adult age (OR: 1.03; 95% CI: 1.01–1.05) and the use of natural freshwater (OR: 2.18; 95% CI: 1.46–3.27) were significant risk factors for *S. haematobium* infection in univariable analyses and remained significant in the adjusted multivariable analyses (n = 1,858).

### Intermediate Host Snail Occurrence and Infection

Between November 2011 and December 2012, a total of 46 freshwater bodies were identified in the 15 selected shehias where MDA plus snail control interventions will be conducted in Unguja. The predominant freshwater bodies were ponds (n = 27), followed by streams (n = 19), mostly of permanent nature. In these water bodies, the average conductivity was 364.6 ms (range: 32–1,657 ms), pH was 8.4 (range: 7.2–12.9), temperature was 29.0°C (range: 24.2–37.9°C), and TDS was 154.3 (range: 15–456). *Bulinus* spp. snails were found in 11 streams (57.9%) and 16 ponds (59.3%). None of the water characteristics was associated with the presence of *Bulinus* spp. None of the *B. globosus/nasutus* shed *S. haematobium* cercariae.

In Pemba, between November 2011 and October 2012, there were a large number of waterbodies (n = 146) identified in the 15 study shehias. The average values of the water chemistry were as follows: conductivity: 298.5 ms (range: 24.4–1,064 ms), pH: 7.1 (range: 4.7–9.4), temperature: 29.6°C (range: 23.0–36.7°C), and TDS: 150.9 (range: 11.2–810). *Bulinus* spp. snails were found in 49 water bodies (33.6%) and their presence was significantly associated with temperature (OR: 1.15; 95% CI: 1.03–1.29) in univariable regression analysis. In total, four *B. globosus/nasutus* from three different sites shed *S. haematobium* cercariae.

### Community Perceptions of Schistosomiasis

FGDs were conducted with 16 groups of primary and 13 groups of secondary school children (n = 150), and with five groups of community members (n = 47). Additionally, IDIs were conducted with 21 teachers, 16 parents, and 12 community leaders.

Most people we talked with associated *kichocho* with urinating blood because of spending time in a dirty river. Despite this association, only a few students and teachers could describe the transmission cycle associated with a parasite and snail. Standing in someone else's urine when in a latrine, stepping in someone's urine in the bush, witchcraft and hexes, walking in dirt infected with *kichocho* organisms, eating chilies, and sexual intercourse between a man and woman were all described as ways to get *kichocho*.

Symptoms of *kichocho* were most often described as abdominal pain, itching of one's private parts with severe pain during urination and bloody urine. Importantly, most people characterized *kichocho* as a boy's disease rather than a girl's disease. Even women and girls believed it to be a disease of boys. One female community member reported, *“I don't know which symptoms are for male and which symptoms are for female. I think it is only a disease of boys.”* Parents knew the least about the transmission of *kichocho*.

People reported that some people self-treated with plant-based teas (often the root of a plant), by drinking lots of water to flush the system, or fail to seek treatment because of anticipated costs. A teacher told us, *“There are some kind of roots made into teas which are used by people (…).* Kichocho *can be treated by these roots.”* Some people told us *kichocho* treatment was free and some described paying for treatment. Even if treatment was free, the cost of transportation was reported as a barrier to seeking care. People also described negative interactions with hospital staff and the lack of available drugs when arriving for treatment at their local health care facility as barriers to seeking care.

People reported that children urinating in the river was a practice contributing to the risk of *kichocho* transmission. Boys were identified as engaging in the riskiest behaviors for acquiring *kichocho* – playing in the river, fishing, and swimming. Young girls were considered at greater risk than older girls for getting *kichocho* because they often played in the river as they had not reached the stage of modesty associated with their culture. People also identified washing clothes in the river as a major risk behavior for both, boys and girls.

When asking the participants for their ideas for preventing children from urinating in the river and spreading schistosomiasis, many people suggested fear and punishment to change urination behaviors and prevent children from going to the river, while at the same time admitting that these methods rarely work. Some people also expressed the need for the community to work together against the disease. A few people reported that it was the role of the government to handle the problem. We were told, *“The best option is give education to our children. But we have nothing in the schools to teach. We need teaching materials and curricula.”* Most people described some ideas for educational, behavioral, or structural interventions to prevent *kichocho* in children.

## Discussion

Elimination of schistosomiasis is now being considered in different parts of the world, including Brazil, the WHO Western Pacific Region, and several countries of the African Region [4]. After careful consideration, SCORE has selected the Zanzibar archipelago to evaluate what intervention combinations are needed to eliminate urogenital schistosomiasis. Lessons from this program will be documented so that elimination programs elsewhere in Africa can benefit. We presented the results from baseline surveys carried out at the onset of a 5-year randomized multi-faceted intervention trial. Hence, the data presented here can serve as a benchmark for monitoring progress as the program unfolds.

In Unguja, we found an overall *S. haematobium* prevalence below 3% in adults and below 5% in school children. Heavy infection intensities in egg-positive school children were rare (15%). In Pemba, the overall prevalence of *S. haematobium* was considerably higher than in Unguja: 6% in adults and 10% in school children, with heavy infections detected in 36% of egg-positive school children. Our data mean that the study objective of “controlling schistosomiasis throughout Pemba island (prevalence <10%) in 3 years” is achieved at this point in time. However, we found considerable heterogeneity. Indeed, we identified a number of “hot-spot” communities on both islands, where the prevalence of *S. haematobium* was above 20% in adults and school children. Population groups with higher odds for *S. haematobium* infections were, besides school children, adult males, younger adults, people born in Pemba or mainland Tanzania, and individuals using natural freshwater.

The existence of some of the hot-spots for schistosomiasis transmission on both, Unguja and Pemba islands is known from previous studies [31], [32] and it seems they have been resilient to the preventive chemotherapy campaigns over the past years, maintaining high prevalences and infection intensities. It will therefore be important to intensify control interventions particularly in these communities in future years to reduce significantly and to interrupt transmission. Future surveillance of the recrudescence of the disease will need to pay special attention to ex-hotspots. It is also widely acknowledged that school boys or adolescents, people using natural freshwater, and those pursuing specific occupations that expose them to open freshwater bodies (e.g., rice farmers) are at an elevated risk of *S. haematobium* infection [16], [33]–[36]. A new and interesting finding of our study is that adult immigrants from mainland Tanzania showed higher odds of *S. haematobium* infection than their counterparts from Zanzibar. Reasons might be arriving from a highly endemic area of schistosomiasis, a different behavior, or a lack of acquired immunity and increased susceptibility to *S. haematobium* infection. For example, immigrants might not have the same exposure history as the local population and could therefore not develop the same level of resistance. A similar observation was made in lifelong residents and residential newcomers in Kenya [37]. It might also be that as *S. haematobium* species are more genetically diverse in Zanzibar than in mainland Africa [38], immigrants might be exposed to *S. haematobium* genotypes to which they are more susceptible. Noteworthy, a study on the neighboring Mafia Island found that all children with a *S. haematobium* infection reported a travel history to mainland Tanzania [39]. Since there is no evidence of active *S. haematobium* transmission on Mafia Island, these cases were almost certainly imported. Therefore, in future surveys, it will be important to determine the influence of migration on the study outcomes, and special attention will be needed on the monitoring of immigrants and potentially imported *S. haematobium* infections from mainland Africa, as well as between Pemba and Unguja.

Prevalence of *S. haematobium* in Pemba has consistently been reported higher than in Unguja [40], [41], most likely due to social-ecological contexts. From our questionnaire surveys, we found that less shehas in Pemba than in Unguja reported the establishment of new clean water sources and latrines in the respective shehia in 2011. In Pemba more people (>75%) than in Unguja (<30%) reported the use of natural freshwater for washing or bathing. This might be due to the fact that Pemba has an undulating landscape with many creeks and streams available in close proximity to houses, while Unguja has a relatively flat terrain with a comparatively low number of streams [42], but also due to the lower availability of artificial clean water sources in Pemba due to its lower economic development [43], [44]. The monitoring of improvements in the water and sanitation infrastructure and the use of clean water and latrines by the people will be essential to adjust correctly our future analyses on the impact of our interventions on *S. haematobium* prevalence and intensity for confounders.

Encouraging for our aim to interrupt schistosomiasis transmission in Unguja over the next 5 years is that none of the snails in Unguja and only very few of the collected *B. globosus/nasutus* snails in Pemba shed *S. haematobium* cercariae. This observation is in contrast to results obtained in a previous study, where patent *S. haematobium* infections were detected, on average, in 4% of the *B. globosus/nasutus* snails collected in Unguja [25]. However, the season and areas where snails were collected differed, and hence data cannot be readily compared. Whether the prepatent infection level in the snails collected in the present survey between November 2011 and December 2012 is higher than the patent observations, remains to be elucidated with molecular methods using a *Dra*I repeat polymerase chain reaction (PCR) approach [25], [45], [46]. The association of *Bulinus* spp. snail presence with temperature in our study in Pemba and with velocity in a previous study [25], highlights the preferences of the snails for specific climatic conditions and their fluctuation in dependence of the rainy season [47], [48]. Given a higher velocity and colder temperature of freshwater in or shortly after the rainy season, we suggest that molluscicides should be applied after the rains have ceased, but before the non-permanent water bodies start to dry out. Snail control will be best harmonized with MDA and ideally be implemented before the population is treated with praziquantel to minimize the risk of rapid re-infection [49].

The qualitative research using FGDs and IDIs showed that, despite Unguja and Pemba having a history of more than 20 years of schistosomiasis control using preventive chemotherapy and, to some extent, health education booklets [50]–[52], the communities' knowledge of disease transmission is only rudimentary. This finding is in line with studies conducted elsewhere in Africa, where poor knowledge on the causes of schistosomiasis was observed [53]–[55]. The behaviors that emerged from our formative research as most important to change in order to reduce schistosomiasis transmission in Zanzibar can be summarized as follows: (i) children urinating in streams and ponds and (ii) children playing, swimming, and washing laundry in the same streams and ponds. Involving community members in the design and implementation of behavioural change interventions will result in a human-centered design intervention tailored to the cultural and social norms of the community with an increase in the likelihood of adoption of the desired protective and preventive behaviors. Participatory hygiene and sanitation transformation (PHAST) interventions already succeeded in increasing knowledge of communities, and transformed into active prevention of schistosomiasis transmission elsewhere in Tanzania [56].

Our finding that macro- and microhematuria were strongly associated with *S. haematobium* infections in adults and school children on both islands is in line with many previous reports from Zanzibar and elsewhere, where the urine examination with reagent strips is suggested as rapid diagnostic tool to identify high-risk areas and to monitor the impact of MDA with praziquantel [57]–[59]. Despite low prevalence and infection intensities found in our baseline survey, the number of egg counts correlated with the color grading of macrohematuria and microhematuria charts. Hence, both macrohematuria and microhematuria are still valid indicators for the detection of true *S. haematobium* cases, even in settings with a long-term history of consistent preventive chemotherapy as found with Zanzibar, and might be used not only for monitoring the impact of our interventions in Zanzibar, but also for future surveillance and response to avoid the recrudescence or reintroduction of the disease. The overall prevalence (9%) of microhematuria particularly in urines from adults implies that there is still considerable morbidity due to schistosomiasis. Hence, when approaching elimination of schistosomiasis transmission, we must not forget that urogenital schistosomiasis is a chronic debilitating disease that affects the urinary and genital tracts of many people and it may continue to impact on public health after the interruption of transmission [35], [60]–[63].

The observation of a higher *S. haematobium* prevalence in first years students compared to 9- to 12-year-old children that attended standards 3 and 4 most likely reflects the impact of praziquantel treatment administered over the past years to school children, but not to pre-school aged children, in school-based treatment programs. Preventive chemotherapy campaigns, and likely also improvements in the sanitary infrastructure, have reduced schistosomiasis prevalences from very high levels (>50%) in the 1980s to today's low level [18], [40], [52], [64].

The low prevalence and intensities of *S. haematobium* infection detected on the Zanzibar islands support our aim to achieve elimination of urogenital schistosomiasis. We must be aware, however, that in addition to the application of MDA, snail control, and behavior change interventions to communities, there will be a need for specifically tailored and sustained control measures to target hot-spot areas, high-risk groups, and individuals with acute and chronic schistosomiasis to achieve and sustain elimination [10]–[12], [65]. Regular assessment of the efficacy of praziquantel and niclosamide will be essential to detect potential resistance development in humans and snails. Finally, to enhance an effective surveillance-response platform, rigorous monitoring, reporting and management of new cases in all health facilities will be essential to avoid a reintroduction of the disease by immigrants and travelers. It is evident, that the human and financial resources needed to eliminate a disease, including schistosomiasis, are considerable. Funds to provide multiple integrated intervention techniques to seriously address schistosomiasis are yet out of reach for most endemic countries. Therefore, it will be important that the governments, institutions, organizations and people from endemic and wealthy countries take responsibility and combine their forces and resources to jointly tackle the last mile towards elimination.

## Supporting Information

Supporting Information S1 **Translation of abstract into language German by author Stefanie Knopp.**
(DOC)

Supporting Information S2 **Study protocol published in BMC Public Health.**
(PDF)

Supporting Information S3 **STROBE table checklist.**
(DOC)
