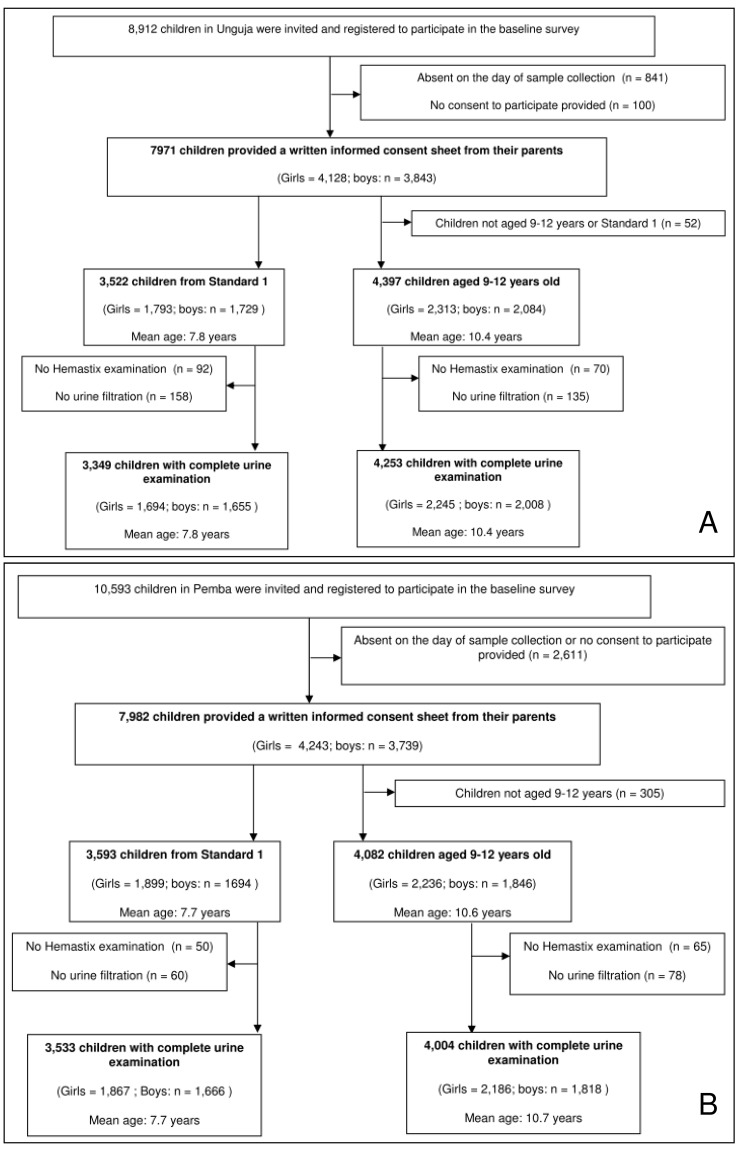# Correction: Elimination of Schistosomiasis Transmission in Zanzibar: Baseline Findings before the Onset of a Randomized Intervention Trial

**DOI:** 10.1371/annotation/d5135174-e4ff-44c6-9c04-17c072a4fd0b

**Published:** 2013-10-22

**Authors:** Stefanie Knopp, Bobbie Person, Shaali M. Ame, Khalfan A. Mohammed, Said M. Ali, I. Simba Khamis, Muriel Rabone, Fiona Allan, Anouk Gouvras, Lynsey Blair, Alan Fenwick, Jürg Utzinger, David Rollinson

The image used for Figure 1 is incorrect. The correct image can be found at the following link: 

**Figure:**