# Supplementary material for: Elimination of Schistosomiasis Transmission in Zanzibar: Baseline Findings before the Onset of a Randomized Intervention Trial
Source: PLoS Negl Trop Dis. 2013 Oct 17;7(10):e2474. doi: 10.1371/journal.pntd.0002474 (PMC3798599; doi:10.1371/journal.pntd.0002474)
Supplement: Supporting Information S1 — Translation of abstract into language German by author Stefanie Knopp. (DOC) [file pntd.0002474.s001.doc]

**Elimination of Schistosomiasis Transmission in Zanzibar: Baseline Findings before the Onset of a Randomized Intervention Trial**

**Elimination der Schistosomiasis Übertragung in Sansibar: Befunde vor dem Beginn einer randomisierten Interventionsstudie**

**Zusammenfassung**

***Hintergrund:*** Die Kontrolle über Schistosomiasis zu erlangen und aufrecht zu erhalten und die Erkrankung, wo immer möglich, regional auszurotten sind Ziele der WHO für das Jahr 2020. In Sansibar haben nationale und internationale Organisationen ihre Kräfte vereint um urogenitale Schistosomiasis innerhalb der nächsten 5 Jahre zu eliminieren. Wir präsentieren Befunde vor Beginn einer randomisierten Interventionsstudie, die dazu konzipiert ist, die unterschiedlichen Auswirkungen von Medikamentenbehandlungen der ganzen Bevölkerung, Schneckenkontrolle und Verhaltensänderungsmaßnahmen zu untersuchen.

***Methoden:*** Anfang 2012 wurde eine parasitologische Grundlagenstudie mit ~20,000 Leuten aus 90 Gemeinden in Unguja und Pemba durchgeführt. Risiko-Faktoren für Schistosomiasis wurden mittels eines Fragebogens für Erwachsene untersucht. In ausgewählten Gemeinden wurde das örtliche Wissen über Schistosomiasis Übertragung und Abwendung durch Fokusgruppen Diskussionen und eingehende Interviews erfasst. Zwischenwirtsschnecken wurden gesammelt und auf das Abgeben von Zerkarien überprüft.

***Wichtigste Ergebnisse:*** Die zu Grunde liegende *Schistosoma haematobium* Prävalenz in Schulkindern und Erwachsenen war 4.3% (Reichweite: 0-19.7%) und 2.7% (Reichweite: 0-26.5%) in Unguja und 8.9% (Reichweite: 0-31.8%) und 5.5% (Reichweite: 0-23.4%) in Pemba. Starke Infektionen wurden in 15.1% und 35.6% der positiven Schulkinder in Unguja and Pemba gefunden. Männer hatten ein grösseres Infektionsrisiko als Frauen (Odds Ratio (OR): 1.45; 95% Vertrauensintervall (CI): 1.03-2.03). Jüngeres Erwachsenalter (OR: 1.04; CI: 1.02-1.06), Geburt in Pemba (OR: 1.48; CI: 1.02-2.13) oder auf dem tansanischen Festland (OR: 2.36; CI: 1.16-4.78), und der Gebrauch von Flusswasser (OR: 2.15; CI: 1.53-3.03) stellten Infektionsrisiken dar. Die Gemeinden hatten ein geringes Wissen über Schistosomiasis. Nur wenige infizierte *Bulinus* Schnecken wurden gefunden.

***Schlussfolgerung/Bedeutung:***

Die niedrige *S. haematobium* Prävalenz in Sansibar ist ein vielversprechender Ausgangspunkt für die Elimination. Es ist jedoch wichtig, dass das Wissen der Bevölkerung über Schistosomiasis Übertragung und Vermeidung verbessert wird. Kontrollmassnahmen, die an lokale Begebenheiten angepasst sind, sind nötig. Wenn Elimination erreicht werden soll, muss besonders auf Hochinfektionsgebiete, Hochrisikogruppen und Einzelpersonen geachtet werden.

***Übersetzung:*** Stefanie Knopp
